# Micropropagation of *Tricholepis roylei* Hook. F.—a point endemic species of the Western Himalaya

**DOI:** 10.1186/s43141-020-00051-9

**Published:** 2020-08-10

**Authors:** Giriraj Singh Panwar, Bhavana Joshi

**Affiliations:** Botanical Survey of India, Northern Regional Centre, Dehradun, Uttarakhand 248195 India

**Keywords:** Habitat rehabilitation, Micropropagation, Point endemic, Species recovery, Threatened

## Abstract

**Background:**

*Tricholepis roylei* Hook. f. is a bushy annual shrub of the Asteraceae family and point endemic species of the Western Himalaya. *T. roylei* is a critically endangered species and factors like poor seed germination and habitat destruction are further rendering the species towards extinction. Therefore, the present investigation was designed to document the seed germination potential of the species and to establish a reproducible in vitro propagation and mass multiplication protocol for the ex situ conservation of *T. roylei* germplasm.

**Results:**

Seeds of *T. roylei* were collected from Sangdha, Himachal Pradesh, India, and were sown in different substrates under open and controlled conditions. Though the overall seed germination potential of the species was reported to be very low and maximum 45% germination was observed in cocopeat substrate after 26 days of incubation. Half-strength Murashige and Skoog (MS) medium enriched with 6-benzylaminopurine (4.4 μM l^−1^) and naphthalene acetic acid (1.0 μM l^-1^) was observed to be the optimum medium for shoot induction in shoot tip explants of *T. roylei*. Maximum 98.89% shoot formation was observed with 28.42 shoots per culture and 4.4-cm shoot length, respectively. The healthy shoots (4.0 cm) were transferred onto rooting media (1/4, 1/2 and full MS) and roots were developed after 8 weeks of incubation in the half-strength MS medium. Half MS augmented with 4.9 μM l^−1^ indole butyric acid was observed to be optimum for the root development and an average of 10.2 roots per shoot with 4.0-cm length was obtained. Rooted plantlets were successfully acclimatized under greenhouse conditions and subsequently established in the field, with a recorded survival rate of 90%. The plants acclimatized to the open environment were also planted in the wild under the habitat rehabilitation and species recovery programme.

**Conclusion:**

The seed germination study envisages that the seed germination potential of the species is very poor and might be one of the probable factors responsible for the shrinkage of *T. roylei* population in the wild. The standardized micropropagation protocol can be helpful for the ex situ conservation of germplasm and rehabilitation of species in the wild. Moreover, the study could be helpful in elucidating the phytochemical and molecular analysis of species.

## Background

*Tricholepis roylei* is a bushy, annual shrub of the Asteraceae family and scattered on rocky habitat all along the roadside. It is a poorly known species of the genus *Tricholepis* and reported in few literatures of India [1]. Due to its poor occurrence in the wild, Hajra [2] has omitted the species from Indian flora and reckoned the species as extinct from the Western Himalaya. Owing to its restricted occurrence (area of occupancy) and few individuals (very small population size) in the wild, the taxon falls under the IUCN designated critically endangered category and is facing the risk of extinction in the imminent future.

Moreover, due to lack of available literature, it is still obscure whether the species is neo-endemic (i.e. taxa that have evolved relatively recently and may be restricted in their distribution because they have not had yet time to disperse further) or paleoendemic (i.e. taxa that have a long evolutionary history and usually are restricted by barriers to dispersal or by extensive extinction in the remaining areas where they were distributed in the past) [3]. Lack of occurrence of the taxon in any botanical literature (state or country flora) and herbaria envisages that the species is neo-endemic in nature and appears very young in the evolutionary time scale.

Due to its point endemic nature and roadside habitat, the species is much prone to anthropogenic disturbances such as massive mining and widening of roads prevalent in the area. Besides this, the Himalaya is one of the most fragile ecosystems on the earth and steep slopes, poor soil binding and heavy monsoon rains further make it vulnerable for habitat destruction, cloud burst, landslide and other natural calamities [4]. Plant species endemic to the Himalayan ecosystem, particularly the point endemic one, are much prone to such factors and might get washed away if any eventuality occurs, which leads towards the extinction of taxa. The situation is further aggravated by the poor seed germination in *T. roylei* revealed by the recruitment of few seedlings in the wild during a personal visit of the area.

The increasing anthropogenic activities in the fragile Himalayan ecosystem are a key driver of the loss of biodiversity in the region [5]. Habitat destruction and overexploitation have resulted in the extinction of umpteen number of plant species and losing a species has grave ecological consequences [6]. All these activities in the region have led to a considerable depletion of the species from the wild. The poor seed germination and continuous habitat degradation may render the species towards extinction in the imminent future. Thus, it is pertinent to conserve the taxon and in vitro technique offers a significant alternative for the rapid mass multiplication and conservation of threatened and endemic species germplasm [7–9].

Genus *Tricholepis* is very significant in terms of its medicinal potential and various species are being utilized as a traditional medicine for the treatment of inflammation, leucoderma and skin diseases. These are also used as a nervine tonic and aphrodisiac and are used in seminal debility [10, 11] malaria, fever, skin grains, stomach pain, blood purification and dysentery [12]. Maher et al. [12] have also isolated two withanolide glucosides (trichosides A and B) from *T. eburnean* with antitumour, antibacterial, antifungal, anti-inflammatory, cytotoxic, hepatoprotective and immunosuppressive activities. Considering the lack of scientific studies and the threatened and point endemic status of the species, the present investigation aimed to study the seed germination potential of the species in different substrates and to standardize micropropagation protocol for the mass multiplication and ex situ conservation of *T. roylei* germplasm by using shoot tip explants.

## Methods

### Collection of seeds and evaluation of seed germination percentage

Mature seeds of *T. roylei* were collected from Sangdha, Himachal Pradesh (2300 masl, 30° 24 N, 78° 17 E), in November 2018. The plant material was identified by the first author with the help of a herbarium specimen available in the BSD and live specimen growing in the Experimental Botanical Garden of Botanical Survey of India, Dehradun, India. Seed germination was studied in both conditions, i.e. field and controlled conditions. Initially, the mature healthy seeds were segregated into two lots and seeds of one lot were washed in running tap water for 30 min. Thereafter, seeds were surface sterilized with 0.1% HgCl_2_ (Himedia Laboratories, Mumbai, India) for 5 min and seeds were thoroughly rinsed with autoclaved double-distilled water. Seeds of one lot were sown in soil, sand and cocopeat and placed in polyhouse at 25 °C. Another lot of disinfected seeds were sown in filter paper and half- and full-strength Murashige and Skoog medium [13] and were kept in the seed germinator (CHM-16, REMI, India) at 25 °C temperature, 50% relative humidity and 16/8-h light and dark photoperiod.

### Initiation of aseptic culture

Young meristematic shoot tip explants of *T. roylei* were collected from the Experimental Botanical Garden of the Botanical Survey of India, Dehradun, and washed in running tap water for 30 min. It was followed by shaking in Tween-20 solution (Himedia Laboratories, Mumbai, India) for 20 min on a rotatory shaker at 100 rpm. Each treatment is followed by thorough rinsing in running tap water to remove the detergent completely. Thereafter, shoot tip explants were surface sterilized with 70% ethanol, 6% NaOCl (Merck & Co., United States) and 0.1% HgCl_2_ (Himedia Laboratories, Mumbai, India). After each treatment, explants were thoroughly rinsed with autoclaved double-distilled water. Properly disinfected explants were inoculated onto Murashige and Skoog (MS) medium enriched with different plant growth regulators (PGRs). Cultures were maintained in the culture room at 24 ± 2 °C, under a 16/8-h light and dark cycle with a light intensity of 47.29 μmol m^−2^ s^−1^ provided by white fluorescent LED tubes (40 W; Wipro, India). All the PGRs used were procured from Himedia Laboratories, Mumbai, India, and glasswares used were from Borosil, India.

### Shoot induction and proliferation

Excised shoot tip explants (1.5–2.0 cm) were inoculated onto basal MS medium (control) and MS medium augmented with different concentrations of cytokinins viz*.* 6-benzylaminopurine (BAP) (2.2 to 6.6 μM), N-phenyl-N0-1,2,3-thiadiazol-5-urea (thidiazuron/TDZ) (2.27 to 6.8 μM) and Kinetin (3.25 to 9.3 μM). Subsequently, the optimal concentration for shoot induction of BAP (4.4 μM), TDZ (4.54 μM) and kinetin (6.9 μM) was further tested in combination with different concentrations of naphthalene acetic acid (NAA) (0.53–1.59 μM).

After shoot initiation, shoot proliferation was performed in modified MS medium (half-strength MS and 5.0 g agar) with optimized hormonal combinations. Shoot proliferation cultures were sub-cultured at a regular interval of 3 weeks. Shoot multiplication rate was calculated on the basis of percentage of explants with a positive response, number of total shoots per explant and shoot height after 6 weeks of incubation.

### Root induction and hardening

After 6 weeks of culture, the in vitro-regenerated healthy shoots (3–4 cm in length) were transferred to root induction media, i.e. MS and modified MS medium (quarter and half salt-strength). Later, the optimal medium was augmented with different concentrations of auxins viz*.* indole butyric acid (IBA) (2.46 to 7.36 μM), indole acetic acid (IAA) (2.85 to 8.56 μM) and NAA (2.65 to 7.9 μM). Cultures were incubated under the same conditions as above and rooting percentage, number of roots and root length were recorded after 8 weeks of incubation.

Plantlets with properly developed shoot and root were taken out from the culture flasks after 6 weeks of incubation and washed gently under running tap water to detach the traces of the medium from the roots. Plantlets were shifted to poly bags containing sand and were placed in the polyhouse for 1 month. Plantlets were provided half-strength modified Hoagland solution [14] at 3-day interval. In order to acclimatize plants to field conditions, plantlets were transferred to pots containing compost-enriched soil after 4 weeks and maintained in the greenhouse.

All the experiments were conducted in triplicates and each set of experiment was carried out with 20 seeds/explants. Analysis of variance and mean separation was carried out using Duncan’s multiple range test (DMRT) utilizing SPS software.

## Results

### Seed germination

**Fig. 1:**
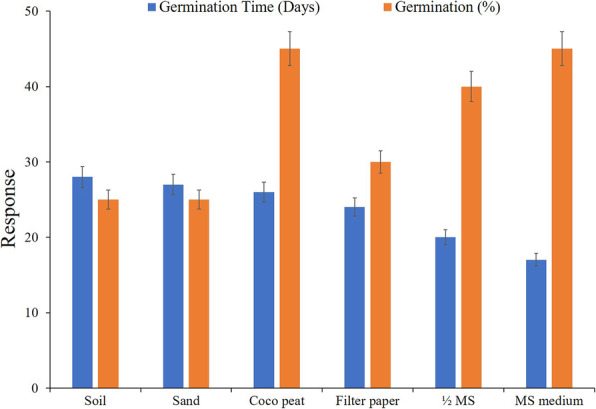
Seed germination response of *Tricholepis roylei* in different substrates

Observation on seed germination experiments, sown in different substrate viz*.* soil, sand, cocopeat, filter paper, 1/2 MS and full MS medium revealed that the germination percentage of *T. roylei* seeds was very low (25–45%) in all the substrates used. Cocopeat was found to be the best substrate for the seed germination and maximum 45% germination was observed after 26 days of incubation. Seeds sown in 1/2 MS and basal MS medium exhibit 40% germination after 17 days of incubation. Soil and sand exhibit minimum seed germination (25%) with the maximum incubation period of 28 and 27 days, respectively (Fig. 1).

### Initiation of aseptic culture and shoot induction

**Fig. 2:**
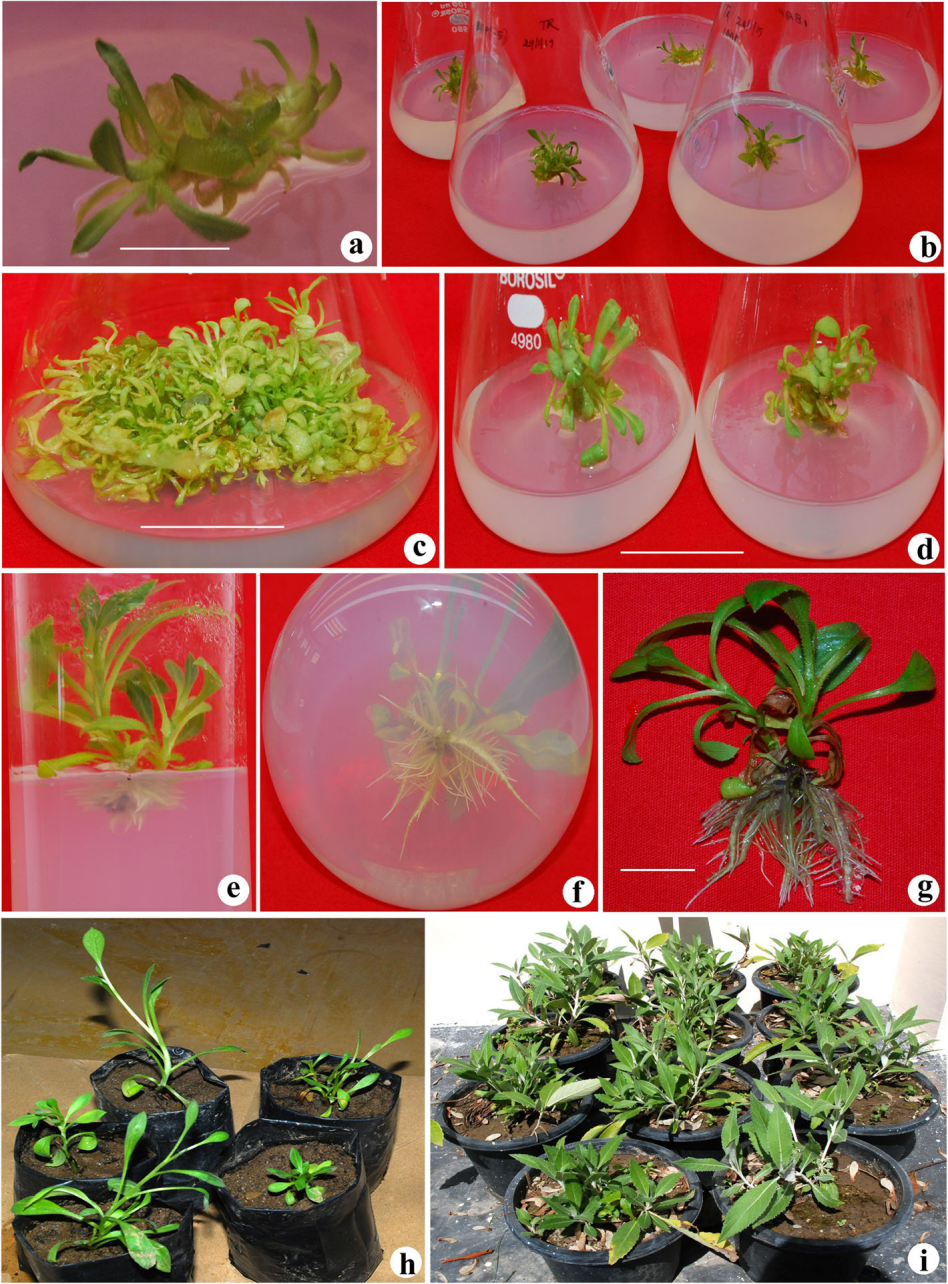
Micropropagation of *Tricholepis roylei*. **a**, **b** Initiation of shoots from shoot tip explants after 1 week of incubation in half-strength MS medium enriched with BAP (4.4 μM) plus NAA (1.0 μM). **c** Proliferation of shoots in half-strength MS medium containing BAP (4.4 μM) plus NAA (1.0 μM). **d** 6-week-old shoots shifted to half-strength rooting medium. **e** Initiation of roots after 3 weeks of incubation in half-strength MS medium containing IBA (4.9 μM). **f**, **g** Plantlet with fully developed roots after 8 weeks of incubation. **h** Hardening of plantlets in plastic bags containing sand. **i** Fully acclimatized plants transferred to pots containing compost-enriched soil after 4 months. (Bars represent 2 cm)

The excised shoot tip explants were inoculated onto the shoot initiation basal MS and half-strength MS medium (control). Explants inoculated onto control medium did not show any morphogenic response. When both the basal and half-strength media were enriched with BAP (2.2, 3.1, 4.4 and 6.6 μM), TDZ (2.27, 3.1, 4.5 and 6.8 μM) and kinetin (3.25, 4.6, 6.9 and 9.3 μM) alone, a significant increase was observed in the shoot formation percentage and maximum 68.15% shoot development was observed in BAP-substituted medium followed by TDZ (51.27%) and kinetin (46.29%), respectively (Table 1). Among both the mediums (1/2-strength and full MS), half-strength MS medium exhibits the best response while vitrified shoot cultures were obtained in MS medium. Half-strength MS medium was observed to be optimal for shoot formation; hence, all the further experiments were carried out in the half-strength MS medium. Since BAP (4.4 μM), TDZ (4.54 μM) and kinetin (6.92 μM) yielded the maximum shoot proliferation rate in the half-strength MS medium and were further tested in combination with various concentrations of NAA (0.53–1.59 μM). A remarkable increase was observed in the shoot proliferation rate due to the synergistic effect of both the PGRs (Table 1). Half-strength MS reinforced with BAP (4.4 μM l^−1^) and NAA (1.06 μM l^−1^) was found to be the optimal medium for shoot initiation and proliferation, followed by TDZ (4.5 μM l^−1^) plus NAA (1.06 μM l^−1^) and kinetin (6.9 μM l^−1^) plus NAA (1.06 μM l^−1^), respectively. Based on all the experiments, half-strength MS medium reinforced with 4.4 μM l^−1^ BAP and 1.6 μM l^−1^ NAA was observed to be optimal for the shoot development and proliferation and maximum 98.89% shoot formation was achieved with 28.42 mean shoot number per culture and 4.4-cm shoot length, respectively (Fig. 2a–c). Among cytokinins, BAP was observed as the potent cytokinin for shoot induction as well as shoot proliferation followed by TDZ and kinetin, respectively. The agar concentration of the MS medium also played a significant role in the development of normal shoot in *T. roylei* shoot culture. MS and half MS medium with 6.0–8.0 g agar quantity exhibits reduced shoot number and growth, while augmenting the medium with 5.0 g agar was observed to be optimal and maximum 98.89% shoot growth was observed in half-strength MS medium (Table 2).

**Table 1:** Effect of cytokinins in combination with NAA on shoot proliferation from shoot tip explants of *Tricholepis roylei* inoculated in half-strength MS medium after 6 weeks of culture

Plant growth regulators (μM)	Explants with shoots (%)	No. of shoots per explant	Shoot length (cm)
MS_0_	-	-	-
BAP			
2.2	18.09	5.37 ± 0.9^j^	1.9 ± 0.2^h^
3.1	36.87	10.08 ± 1.2^h^	3.1 ± 0.4^f^
4.4	68.15	15.48 ± 1.6^f^	3.4 ± 0.47^e^
6.6	59.04	13.26 ± 1.2^f^	3.2 ± 0.4^e^
TDZ			
2.27	17.58	5.27 ± 0.8^j^	1.8 ± 0.4^h^
3.18	35.47	10.00 ± 1.1^h^	2.8 ± 0.2^g^
4.54	51.27	13.67 ± 1.3^f^	3.3 ± 0.2^e^
6.8	46.38	11.48 ± 1.0^g^	3.1 ± 0.2^f^
Kinetin			
3.25	13.48	4.98 ± 0.8^j^	1.7 ± 0.6^i^
4.6	34.23	8.89 ± 1.1^i^	2.7 ± 0.3^g^
6.92	46.29	12.55 ± 1.3^g^	3.3 ± 0.2^e^
9.3	41.85	11.07 ± 1.2^h^	3.0 ± 0.15^f^
BAP+NAA			
4.4 + 0.53	82.23	17.32 ± 1.4^e^	4.1 ± 0.6^c^
4.4 + 1.06	98.89	28.42 ± 2.1^a^	4.4 ± 0.9^a^
4.4 + 1.59	95.26	24.55 ± 2.0^b^	4.3 ± 0.8^b^
TDZ+NAA			
4.5 + 0.53	74.82	16.89 ± 1.4^e^	4.1 ± 0.6^c^
4.5 + 1.06	89.01	25.01 ± 2.0^b^	4.3 ± 0.8^b^
4.5 + 1.59	79.72	23.36 ± 1.9^c^	4.2 ± 0.6^c^
Kinetin+NAA			
6.9 + 0.53	68.86	14.44 ± 1.5^f^	3.9 ± 0.5^d^
6.9 + 1.06	80.82	24.18 ± 2.0^b^	4.2 ± 0.6^c^
6.9 + 1.59	77.66	21.05 ± 1.8^d^	4.1 ± 0.6^c^

#Data are presented as the mean ±SD. Means followed by different letter within columns indicate significant differences at *p* ≤ 0.05

**Table 2:** Effect of agar concentration on development of shoots from shoot tip explants of *T. roylei* inoculated onto half-strength MS medium

Agar concentration (g)	Explants with shoots (%)	No. of shoots/culture	Shoot length (cm)
5.0	98.89	28.42 ± 2.1	4.4 ± 0.9
6.0	79.32	17.35 ± 1.0	3.1 ± 0.81
8.0	68.34	9.56 ± 0.9	2.3 ± 0.4

### Root induction

**Fig. 3:**
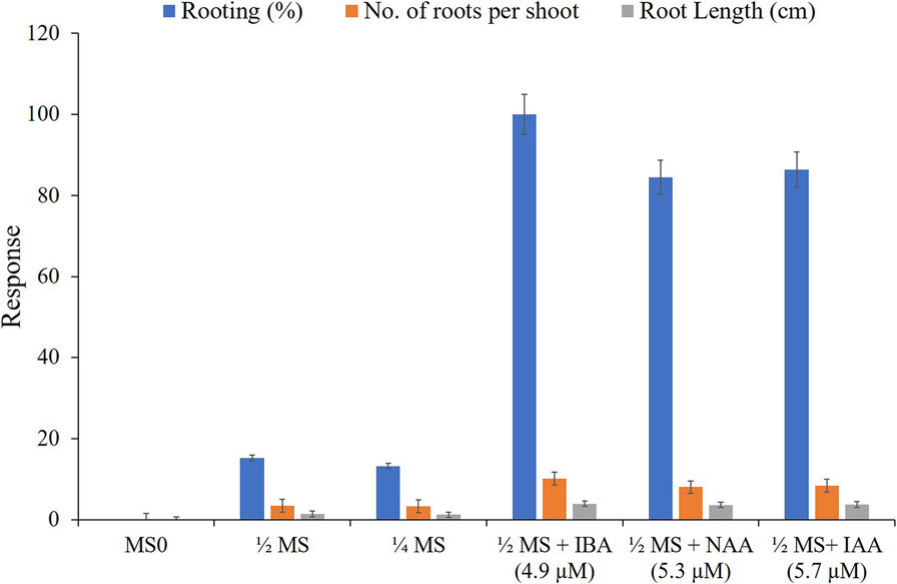
Effect of nutrient strengths of MS medium and auxins on root induction in *Tricholepis roylei*

Properly developed healthy shoots (4.0-cm height) were shifted for the root induction onto basal MS and modified MS medium (half- and quarter-strength). Shoots shifted to basal MS medium did not yield any rooting response, while 15.27 and 13.32% rooting was observed in the half- and quarter-strength MS medium, respectively (Fig. 3). Since half-strength MS medium yielded better morphogenic response, further experiments were conducted in half-strength MS medium. By incorporation of IBA (2.46–7.36 μM), NAA (2.85–8.56 μM) and IAA (2.65–7.9 μM) into half-strength MS medium, a remarkable increase was observed in the rooting percentage. Maximum 100%, 86.47% and 84.54% rooting was observed in IBA, IAA and NAA augmented half-strength MS medium, respectively (Table 3). The half-strength MS medium augmented with IBA (4.9 μM l^−1^) was found to be optimal for root development in *T. roylei* and 100% rooting was achieved with average 10.2 roots per shoot after 8 weeks of incubation (Fig. 2d–g).

**Table 3:** Effect of auxins on root induction in in vitro-regenerated shoots of *Tricholepis roylei* in half-strength MS medium after 4 weeks of culture

Auxins (μM)			Rooting (%)	No. of roots per shoot	Root length (cm)
IBA	NAA	IAA			
MS_0_	0	0	-	-	-
1/2MS_0_	0	0	15.27	3.5 ± 0.3^e^	1.5 ± 0.9^e^
1/4MS_0_	0	0	13.32	3.3 ± 0.3^f^	1.3 ± 0.6^f^
2.46	0	0	72.37	7.8 ± 0.9^d^	3.5 ± 0.4^d^
4.9	0	0	100.00	10.2 ± 1.4^a^	4.0 ± 0.9^a^
7.36	0	0	92.82	9.5 ± 1.3^a^	3.9 ± 0.7^a^
0	2.65	0	68.59	7.7 ± 0.9^d^	3.4 ± 0.7^d^
0	5.3	0	84.54	8.1 ± 0.9^c^	3.7 ± 0.8^b^
0	7.9	0	79.38	8.0 ± 0.9^c^	3.7 ± 0.8^b^
0	0	2.85	69.24	7.9 ± 0.8^d^	3.5 ± 0.7^c^
0	0	5.71	86.47	8.4 ± 1.0^b^	3.8 ± 0.9^b^
0	0	8.56	71.53	8.1 ± 1.0^c^	3.6 ± 0.8^c^

#Data are presented as the mean ±SD. Means followed by different letter within columns indicate significant differences at *p* ≤ 0.05

### Hardening and transplantation

The plantlets with well-developed roots were shifted to poly bags containing sand (Fig. 2h) and were kept inside the greenhouse for 1 month. After 1 month, plants were shifted to pots containing compost-enriched soil in the greenhouse. Fully acclimatized plants were finally transferred to the open field with 90% success (Fig. 2i) and some plants were also transferred to wild habitat under the habitat rehabilitation and species recovery programme.

## Discussion

The seed germination study in *T. roylei* revealed that the seed germination potential of the species is very poor and might be one of the factors responsible for the dwindling population size of the species. Seeds sown in different substrates did not show any significant difference and cocopeat was reported as a suitable substrate for seed germination in *T. roylei,* but the mean germination time was higher (28 days) as compared to in vitro experiments (17 days). Cocopeat was also reported as the best substrate for seed germination studies on *Stereospermum suaveolens* [15], *Oroxylum indicum* [16] and *Gonystylus bancanus* [17].

MS medium enriched with different concentrations of cytokinins (BAP, TDZ and Kin) was used for the shoot induction in shoot tip explants of *T. roylei* and the optimum response was observed in MS medium enriched with BAP. But besides shoot formation, callusing was also reported at the cut base of the explants in BAP-enriched MS medium, thus affecting the shoot induction percentage of shoot tip explants. Reducing the nutrient strength of MS medium to half with similar hormonal compositions ceased the callusing and consequently enhanced the shoot development percentage (68%) in shoot tip explants. Similar report was also observed in *V. amygdalina* and was overcome by reducing the nutrient composition of MS medium to half of the original strength [18]. Among different cytokinins tested, BAP was observed as the most potent cytokinin and significant difference was observed in shoot induction percentage of BAP (68%), TDZ (51%) and kinetin (46.29%). Transcendence of BAP over the other cytokinins was also reported in earlier findings on *Aconitum violaceum* [19] and *Digitalis ferruginea* [20]. Further, augmenting the BAP-, TDZ- and kinetin-enriched half-strength MS media with different concentrations of NAA, a remarkable increase was observed in the shoot multiplication percentage, number of shoots and shoot length. The half-strength MS medium enriched with BAP (4.4 μM l^−1^) plus NAA (1.06 μM l^−1^) was observed as optimal, and maximum 98.89% shoot formation was achieved along with 28.42 mean shoot number per explant and 4.4 cm shoot length. The synergistic effect of both the PGRs (BAP and NAA) significantly enhanced the shoot development percentage as well as mean shoot number per explant. Similar findings were also reported on *Eremostachys superba* [21], *Senecio macrophyllus* [22] and *Rauwolfia serpentina* [23]. The agar concentration of MS medium also played a significant role in the development of normal shoots. MS and half MS medium containing 6.0–8.0 g agar exhibits reduced shoot growth, while reduction of agar quantity to 5.0 g was observed to be optimal for the normal shoot growth in the half-strength MS medium. The effect of agar concentration was also pronounced in a previous finding on *Ceropegia thwaitesii* [24].

Shoots shifted for the root induction in MS and modified MS medium (half and quarter strength), the best response was obtained in half-strength MS medium followed by quarter-strength MS medium, while basal MS medium did not yield any response. Half-strength MS medium enriched with IBA showed the best root formation (100%) followed by IAA (86.47%) and NAA (84.54%), respectively. Half-strength MS medium enriched with IBA (4.9 μM l^−1^) proved to be the optimal and average 10.2 roots per shoot with 4.0-cm root length was obtained after 8 weeks of incubation. The supremacy of IBA over other auxins was also observed in *Lilium polyphyllum* [25] and *Zanthoxylum armatum* [26]. Plantlets fully acclimatized in the polyhouse were finally transferred to the open environment with 90% success.

## Conclusion

In conclusion, the current investigation for the first time documents the seed germination behaviour of the species and also established an efficient, reproducible protocol for the micropropagation of *T. roylei,* a point endemic species of the Western Himalaya*.* This study assured effective establishment, mass multiplication and offering an in vitro strategy for the ex situ conservation of critically endangered and point endemic *T. roylei* germplasm. This study further will be more useful in elucidating the phytochemical pathway/potential of the species.

## Data Availability

Not applicable

## Abbreviations

BAP: 6-Benzylaminopurine
NAA: Naphthalene acetic acid
IBA: Indole butyric acid
IAA: Indole acetic acid
MS: Murashige and Skoog
PGRs: Plant growth regulators
TDZ: N-Phenyl-N0-1,2,3-thiadiazol-5-urea or thidiazuron

## References

[1] Chaudhary LB, Pandey AK (2001). Revision of *Tricholepis* DC. (Asteraceae) in India. Rheedea.

[2] Hajra PK, Hajra PK, Rao RR, Singh DK, Uniyal BP (1995). Carduear. Flora of India.

[3] Morrone JJ, Jorgensen SE, Fath BD (2008). Endemism. Evolutionary ecology. Vol. [2] of encyclopedia of ecology, 5 vols.

[4] Rizvi J (1981). The fragile Himalaya: a review article. India Int Centre Q.

[5] Young RH (2009). Land use and biodiversity relationships. Land Use Policy.

[6] Raffaelli D (2004). How extinction patterns affect ecosystems. Science.

[7] Bhatt ID, Dhar U (2004). Factors controlling micropropagation of *Myrica esculenta* Buch-ham ex D.Don: a high value wild edible of Kumaun Himalaya. Afr J Biotechnol.

[8] Giri L, Dhyani P, Rawat S, Bhatt ID, Nandi SK, Rawal RS, Pande V (2012). In vitro production of phenolic compounds and antioxidant activity in callus suspension cultures of *Habenaria edgeworthii*: a rare Himalayan medicinal orchid. Ind Crop Prod.

[9] Nandi SK, Palni LMS, Pandey H, Chandra B, Nadeem M, Anis M, Ahmad N (2016). Selection of elites and in vitro propagation of selected high-value Himalayan medicinal herbs for sustainable utilization and conservation. Plant tissue culture: propagation, conservation and crop improvement.

[10] Padashetty SA, Mishra SH (2007). Aphrodisiac studies of *Tricholepis glaberrima* with supportive action from antioxidant enzymes. Pharm Biol.

[11] Khare CP (2007). Indian medicinal plants-an illustrated dictionary.

[12] Maher S, Rasool S, Mehmood R, Perveen S, Tareen RB (2018) Trichosides A and B, new withanolide glucosides from *Tricholepis eburnean*. Nat Prod Res 32:1–6 10.1080/14786419.2015.1030340 25868474

[13] Murashige T, Skoog F (1962). A revised medium for rapid growth and bioassays with tobacco tissue cultures. Physiol Plant.

[14] Epstein E (1972). Mineral nutrition of plants: principles and perspectives.

[15] Trivedi D, Joshi AG (2014). Studies on seed germination of *Stereospermum suaveolens* with respect to different parameters. Environ Exper Biol.

[16] Trivedi D, Joshi AG (2012). Effect of substrates on the growth of *Oroxylum indicum* (vent.). Indian Forester.

[17] Utami NW, Witjaksono HDSH (2006). Seed germination and seedling growth of Ramin (*Gonystylus bancanus* Miq.) on various growing media. Biodiversitas.

[18] Joshi B, Bhandari A, Panwar GS (2020) An efficient micropropagation protocol for *Vernonia amygdalina *Delile – an economically valuable shrub. J Herbs Spices Med Plants 26(3):267–274

[19] Rawat JM, Rawat B, Agnihotri RK, Chandra A, Nautiyal S (2013) In vitro propagation, genetic and secondary metabolite analysis of *Aconitum violaceum* Jacq.-a threatened medicinal herb. Acta Physiol Plant. doi.org/10.1007/s11738-013-1294-x.

[20] Verma SK, Yucesan B, Sahin G, Gurel E (2014) Embryogenesis, plant regeneration and cardiac glycoside determination in *Digitalis ferruginea* subsp *ferruginea* L. Plant Cell Tissue Org Cult 119:625–634

[21] Panwar GS, Srivastava SK, Uniyal PL (2015). *In vitro* propagation of *Eremostachys superba* Royle ex Benth. – an endangered, medicinal and ornamental herb of north-west Himalaya. Med Plants.

[22] Trejgell A, Michalska M, Tretyn A (2010). Micropropagation of *Senecio macrophyllus* M. Bieb. Acta Biol Cracov Ser Bot.

[23] Singh G, Guru SK (2007). Multiple shoot induction in intact shoot tip, excised shoot tip and nodal segment explants of *Rauwolfia serpentina*. Indian J Plant Physiol.

[24] Muthukrishnan S, Benjamin JHF, Rao MV (2013). Influence of agar concentration and liquid medium on *in vitro* propagation of *Ceropegia thwaitesii* hook- an endemic species. J Agric Technol.

[25] Panwar GS, Srivastava SK, Uniyal PL (2017). Callus-mediated organogenesis in *Lilium polyphyllum* D. Don ex Royle: a critically endangered Astavarga plant. Curr Sci.

[26] Purohit S, Joshi K, Rawat V, Bhatt ID, Nandi SK (2019) Efficient plant regeneration through callus in *Zanthoxylum armatum* DC: an endangered medicinal plant of the Indian Himalayan region. Plant Biosystems. doi.org:10.1080/11263504.2019.1610107.

